# Emergence of HBV resistance to lamivudine (3TC) in HIV/HBV co-infected patients in The Gambia, West Africa

**DOI:** 10.1186/1756-0500-4-561

**Published:** 2011-12-23

**Authors:** Balint Stewart, Modou L Jobarteh, Ramu Sarge-Njie, Abraham Alabi, Thushan de Silva, Kevin Peterson, Ingrid Peterson, Hilton Whittle, Sarah Rowland-Jones, Assan Jaye, Matthew Cotten, Maimuna Mendy

**Affiliations:** 1 Medical Research Council, Fajara, P O Box 273, Banjul, The Gambia, West Africa; 2 Medical Research Unit, Albert Schweitzer Hospital, Lambarene, Gabon; 3 Weatherall Institute of Molecular Medicine, University of Oxford, John Radcliffe Hospital, Headington, Oxford OX3 9DS, UK; 4 Wellcome Trust Sanger Institute, Hinxton, Cambridge CB10 1SA, UK; 5 International Agency For Research on Cancer (IARC), 150 Cours Albert Thomas, Lyon 69372, France

## Abstract

**Background:**

Lamivudine **(**3TC) is a potent inhibitor of both Hepatitis B virus (HBV) and Human Immunodeficiency Virus (HIV) replication and is part of first-line highly active antiretroviral therapy (HAART) in the Gambia. Unfortunately, the effectiveness of 3TC against HBV is limited by the emergence of resistant strains.

**Aim:**

The aim of this retrospective study was to characterise 3TC-resistant mutations in HBV from co-infected patients receiving HAART, by generating HBV polymerase sequence data and viral loads from HBV genotype E infected patients, both at initiation and during a course of 3TC therapy.

**Method:**

Samples from 21 HBV chronic carriers co-infected with HIV-1 (n = 18), HIV-2 (n = 2) and HIV-dual (n = 1) receiving HAART for a period of 6-52 months were analysed for the emergence of 3TC-resistance mutations.

**Findings:**

Sixteen out of 21 HBV/HIV co-infected patients responded well to HAART treatment maintaining suppression of HBV viraemia to low (≤ 10^4 ^copies/mL) (n = 5) or undetectable levels (< 260 copies/ml) (n = 11). Out of the 5 non-responders, 3 had developed 3TC-resistant HBV strains showing mutations in the YMDD motif at position 204 of the RT domain of the HBV polymerase. One patient showed the M204V^+ ^L180M^+ ^V173L^+ ^triple mutation associated with a vaccine escape phenotype, which could be of public health concern in a country with a national HBV vaccination programme. All except one patient was infected with HBV genotype E.

**Conclusions:**

Our findings confirm the risk of 3TC mutations in HAART patients following monotherapy. This is a novel study on 3TC resistance in HBV genotype E patients and encourage the use of tenofovir (in association with 3TC), which has not shown unequivocally documented HBV resistance to date, as part of first-line therapy in HIV/HBV co-infected patients in West Africa.

**HBV**- hepatitis B infection; **HIV**- human immunodeficiency virus; HA**ART**- antiretroviral therapy.

## Background

Hepatitis B virus (HBV) and human immunodeficiency virus (HIV) both pose significant public health challenges in the developing world. Globally, approximately 10% of HIV- infected individuals are also chronic carriers of HBV [1] as a consequence of shared modes of transmission. In The Gambia, where HBV is endemic the figure is closer to 15%, with HBV infection occurring most commonly before the age of 4 years and usually preceding HIV infection [2,3].

Untreated HIV/HBV co-infection is characterised by higher HBV viral loads [4] and a reduction in the frequency of hepatitis B e antigen (HBeAg) seroconversion to antibody against HBe (anti-HBe), which is predictive of poorer clinical outcomes [5]. HIV/HBV co-infection is associated with higher liver-related morbidity and mortality compared to mono-infection with either virus [6]. With the increasing availability of highly active antiretroviral therapy (HAART) liver disease has become a leading cause of non-acquired immunodeficiency syndrome (AIDS)- related death in HIV/HBV co-infected patients [6].

In 1997 the Food and Drug Administration approved the use of 3TC, an orally-administered nucleoside analogue that potently suppresses replication of both HBV and HIV [7]. Based on WHO guidance, this drug is included in first-line HIV treatment in the Genito-Urinary Medicine (GUM) clinic in The Gambia; and is the only HBV-active drug routinely received by ART-naïve patients. Patient on 3TC monotherapy are at risk of developing resistant mutations following 1-2 years of treatment. Four major patterns of resistance mutations have been described. with the most important occurring in the highly conserved tyrosine-methionine-asparate-aspartate (YMDD) motif, involving a change from methionine at position 204 in the reverse transcriptase domain (rt204) to either a valine or isoleucine residue (rtM204V/I) or, more rarely, a serine residue [8,9].

Compensatory mutations at other sites are usually required to reduce the loss in replication fitness associated with YMDD mutants [10,11]. As a result of significant open reading frame overlap in the HBV genome, changes in polymerase can lead to changes in the surface region which can induce changes in HBsAg secretion with significant reduction in HBsAg antigenicity [12-15].

As a result of the important role played by the secreted hepatitis B surface antigen (HBsAg) in immune recognition following vaccination, mutations in the HBV surface gene can have diagnostic and public health implications for universal vaccination programme.

The aims of this retrospective study was to, characterise 3TC resistance mutations in HBV genotype E chronic carriers receiving HAART.

## Results

From an initial cohort of 31 HIV/HBV co-infected patients on HAART, 9 were excluded from the study either due to doubts about adherence to therapy (n = 2), seroreversion from serum HBsAg positive to HBsAg- negative during the recruitment period, prior to start of treatment (n = 1) or had been on treatment for < 6 months (n = 6). A 10^th ^patient was excluded because the patient's virus had the M204I mutation at the initiation of HAART and further investigation revealed that this patient had previously been on zidovudine AZT/3TC dual therapy when the mutation was likely to have emerged. The baseline characteristics of the remaining 21 patients are summarised in Table 1. The patients were all adults, (> 18 yrs old) and were co-infected with HIV-1 (n = 18), HIV-2 (n-2) or HIV-1 plus HIV-2 (n = 1). Pre-treatment HBV viral loads ranged from undetectable levels (< 260 copies/mL) to 1.0 × 10^9 ^copies/mL. Baseline HBV viral load was high (≥ 10^5 ^copies/mL) in the 6 HBeAg-positive patients and in 7/15 of the HBeAg-negative patients. Patients with high HBV DNA loads tended to have higher alanine aminotransferase and aspartate aminotransferase liver enzyme concentrations. The DNA sequence of all except one patient, were similar to HBV genotype E.

**Table 1:** Baseline profiles and HBV/HIV viral load profile of 21 chronic HBV carriers on HAART

Baseline time point							Treatment				
**Patient ID**	**Age**	**Sex**	**HBeAg**	**ALT/AST**	**HIV type**	**CD4**	**Treatment duration (Months)**	**HIV VL** **Pre HAART**	**HIV VL** **end point**	**HBV VL** **Pre HAART**	**HBV VL** **end point**

	(yrs)			(IU/ml)		Count (/mm^3^)		(c/mL)		(c/mL	
1	42	M	-	33/28	HIV-1	110	25	1.0 × 10^6^	1.0 × 10^2^	6.5 × 10^4^	4.5 × 10^4^
3	37	F	-	32/23	HIV-1	10	25	8.7 × 10^5^	1.0 × 10^2^	2.8 × 10^4^	< 260
5	40	M	-	37/33	HIV-1	990	44	4.7 × 10^2^	1.0 × 10^2^	5.9 x10^5^	2.4 × 10^8^
6	35	F	+	95/39	HIV-1 & 2	300	33	3.6 × 10^4 ^(HIV-1) < 1.0 × 10^2 ^(HIV-2	1.0 × 10^2^ (HIV-1 & HIV-2)	1.0 × 10^6^	3.7 × 10^7^
7	28	F	+	67/43	HIV-1	290	22	7.3 × 10^4^	1.0 × 10^2^	3.5 × 10^7^	1.4 × 10^3^
9	44	M	+	83/25	HIV-1	240	6	2.3 × 10^8^	1.0 × 10^2^	5.4 × 10^8^	1.8 × 10^9^
10	40	M	+	99/5	HIV-1	80	25	1.0 × 10^6^	1.2 × 10^4^	2.3 × 10^8^	6.5 × 10^3^
12	48	M	-	38/21	HIV-1	80	26	1.0 × 10^6^	2.0 × 10^4^	3.0 × 10^7^	7.9 × 10^7^
13	36	M	-	54/22	HIV-1	60	36	4.1 × 10^5^	1.0 × 10^2^	4.6 × 10^7^	2.5 × 10^7^
16	41	F	-	42/12	HIV-2	340	25	1.3 x10^6^	1.0 × 10^2^	1.2 × 10^8^	< 260
17	27	M	-	NT	HIV-1	10	12	7.5 × 10^4^	1.0 × 10^2^	5.0 × 10^5^	< 260
18	50	F	+	NT	HIV-1	210	18	1.8 × 10^5^	1.0 × 10^2^	7.9 × 10^8^	8.1 × 10^4^
19	24	F	+	40/22	HIV-1	160	50	1.6 × 10^5^	1.0 × 10^2^	1.5 × 10^9^	< 260
21	42	F	-	35/24	HIV-2	650	24	1.5 × 10^4^	1.0 × 10^2^	1.7 × 10^4^	< 260
22	29	F	-	42/21	HIV-1	160	24	1.0 × 10^6^	1.0 × 10^2^	1.1 × 10^9^	4.5 × 10^4^
23	52	M	-	38/23	HIV-1	50	50	5.6 × 10^4^	1.0 × 10^2^	5.9 × 10^3^	< 260
24	29	F	-	29/12	HIV-1	220	52	2.4 × 10^5^	1.0 × 10^2^	< 260	< 260
25	31	F	-	27/11	HIV-1	10	36	1.0 × 10^2^	1.0 × 10^2^	1.0 × 10^4^	< 260
26	50	M	-	32/19	HIV-1	130	30	3.0 × 10^5^	1.0 × 10^2^	9.2 × 10^3^	< 260
28	39	F	-	21/4	HIV-1	360	19	4.2 × 10^3^	1.4 × 10^3^	2. 8 × 10^4^	< 260
29	30	F	-	553/397	HIV-1	220	36	5.1 × 10^5^	1.0 × 10^2^	5.7 × 10^8^	< 260

The upper normal limit of ALT and AST liver enzyme levels is 46 international units/ml (IU/mL; NT-samples were not tested for AST and ALT.

The lower limit of detection of real-time PCR was 260 copies/mL (c/mL) and 100 copies/mL for HBV and HIV respectively.

### Response to therapy

The median treatment duration was 25 (range 6 - 52) months. Patients were defined as responders to 3TC therapy if HBV viral loads were reduced to (≤ 10^4 ^copies/ml) and if they showed sustained viraemic suppression throughout the period under study or as non-responders if their viral load remained at high levels throughout the study or showed > 10^4 ^copies/ml at the end of the period of observation.

Sixteen (76.1%) patients responded well to 3TC treatment, achieving sustained suppression of HBV viraemia; 11 of the 16 (68.7%) patients had undetectable HBV DNA; 3 (50%) of the HBeAg-positive individuals (Patients 10, 18 &19) successfully seroconverted to HBeAg antibody positive and 1 of them (Patient 10) achieved loss of HBsAg as well. Two (66.6%) of the 3 who had seroconverted to HBeAg antibody positive had viral load concentrations of 10^4 ^or 10^3 ^DNA copies per mL respectively.

The DNA sequences of 2 patients who did not respond to treatment and maintained HBV viral loads of > 10^4 ^copies/mL were similar to the wild-type sequence and not the 3TC-resistance mutations.

All, except 3 of the co-infected patients on HAART (# 9, 10 & 28) achieved reduction in HIV viral load (≤ 1.0 × 10^2 ^copies/mL) during the period of the study. Two of these patents were HBeAg positive at the start of therapy. Despite maintaining high levels of HIV RNA (1.0 × 10^4 ^copies/mL), one of them (#10) responded well to HBV treatment; achieving HBsAg clearance, and displaying HBeAg seroconversion and reduction in HBV viral load from 10^8 ^copies/mL to 10^3 ^copies/mL whilst the other patient (#9) maintained high levels of both HIV RNA (10^4 ^copies/mL) and HBV DNA (10^9 ^copies/mL).

### HBV-resistance mutations

**Figure 1:**
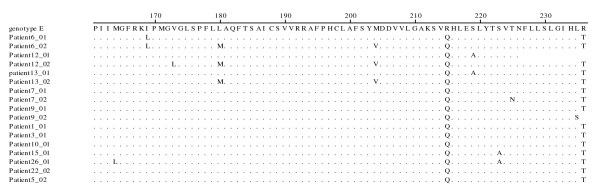
**Amino acid sequence alignment generated from samples at baseline and at most recent time points covering amino acids 161 - 236 of the HBV reverse transcriptase domain**. The HBV RT sequences are aligned with reference genotype E sequence (X75664) which is shown at the top, alignment with patient sequence is shown below with identities marked as (.) and sequence changes indicated by letter. Sequences from patients who developed YMDD mutations (patients 6, 12 and 13) during 3TC therapy are shown at the top half of the figure. The baseline and post treatment time points are represented by '01' and '02' respectively.

Alignment of the HBV RT sequences is shown in Figure 1. HBV resistance to 3TC was found in 3 of 21 (14.2%) co-infected patients including the one patient (#6) with HIV-dual infection who also tested positive for HBeAg (Tables 1 and 2). The remaining 2 patients with 3TC-resistance mutations (# 12 & 13) were infected with a single HIV-1 strain. Patients with 3TC mutations had HBV viral load ≥ 10^4 ^copies/mL throughout the period of observation.

**Table 2:** Patterns of mutation found in patients who developed YMDD 3TC resistant mutants

Patient ID	3TC Mutations			Time after initiation of LMV therapy (months)
Patient 6	M204V	L180M		25
Patient 12	M204V	L180M	V173L*	16
Patient 13	M204V	L180M		36

Times given for when mutation occurred correspond to the first sample given during therapy in which resistance mutations were detected.

*V173L mutation occurred after 23 months from initiation of therapy.

Figure 1 shows the amino acid sequence alignment of the HBV reverse transcriptase region surrounding the YMDD motif at the start of 3TC treatment and at the most recent time points. The M204V mutation was always accompanied by the mutation L180M. The virus from patient 12 had an S219A mutation, which reverted to the wild-type when 3TC resistance mutations V173L, L180M and M204V were acquired. Phylogenetic analysis of the HBV polymerase gene sequences showed close similarity with HBV genotype E, except in the case of 1 patient whose sequences were similar to HBV genotype A (data not shown).

## Discussion

We retrospectively studied 21 HBV-HIV co-infected individuals on HAART to determine the patterns of 3TC resistance mutations. Apart from studies on the immune response to HAART treatment, we are not aware of any published studies on 3TC mutations from West Africa, a region where HBV genotype E infection predominates [16]. In our study, 11 out of 21 patients (52.3%) displayed undetectable viral load at the last point of data collection. The remaining 5 patients had high viral load at the last observation point including 3 patients who had seroconverted to anti-HBe. HBeAg seroconversion usually confers a favourable outcome, however, HBV pre-core (PC) and basal core promoter (BCP) mutations should be excluded because of the clinical implications which could result from enhanced HBV replication or abrogation of HBeAg translation [17].

The observation of patient testing negative for HBsAg at the last point of observation should also be interpreted with caution, since the apparent loss of HBsAg may be due to a poor sensitivity and/or specificity of the assay in detecting mutation in the 'a' determinant region of HBsAg.

Because of the variation in the period of patient observation, it will be difficult to acurately define the rates of the events of HBeAg seroconversion or HBsAg clearance.

Despite its high efficacy, 3TC is not recommended for use as HBV monotherapy in HIV/HBV co-infected patients because of the risk of emergence of resistance mutations in HBV [18]. Similarly, the European AIDS Clinical Society guidelines for treatment of HBV/HIV co-infected patients recommend that patients with no indication of anti-HIV treatment should not be treated with drugs such as 3TC, entacavir and tenofovir because of the risk of developing HIV-resistant mutations [19]. However, the criteria for treatment for the patients in the study were based solely on their HIV status.

Although HIV-1 and HIV-2 infections currently show similar prevalence in The Gambia, patients infected with HIV-2 are far less likely to progress to AIDS than patients infected with HIV-1 [20]; thus when considering all HIV-1 and HIV-2 patients on HAART, there is a bias towards HIV-1 infections.

The introduction of 3TC-containing HAART is a relatively recent development in The Gambia, and therefore, 3TC-resistance mutations were not observed in therapy-naïve individuals in the present study. However, with the continued use of 3TC as the only HBV-active drug in HAART patients, the proportion of resistant strains may increase with time, which will significantly reduce HBV treatment effectiveness [21].

Thus due to the potential association of the triple 3TC mutations (M204V^+ ^L180M^+ ^V173L^+^) with vaccine escape mutants, the continuous surveillance of resistance mutants is necessary in a country with a national HBV vaccination programme [22,23]. Additionally, with the decline in HIV-associated morbidity and mortality following the introduction of HAART, there is a need for screening of HIV patients for underlying viral hepatitis co-infection and the provision of management and treatment recommendations for patients with chronic viral hepatitis in preventing the development of liver disease.

## Materials and methods

### Subjects

Five hundred and seventy HIV-positive individuals were screened for hepatitis B markers. Seventy (12.2%) of them were HBsAg-positive and 31 were receiving HAART that included 3TC (300 mg/day). Samples from 21 out of the 31 co-infected patients receiving HAART were analysed retrospectively. Six (28.5%) of the 21 patients tested positive for HBeAg, 18 (85.7%) for HIV1, 2 (9.5%) for HIV-2 and 1 for HIV-dual infections. The HIV viral load ranged from undetectable to 1.1 × 10^9 ^(Table 2).

Seventeen of these patients had received 3TC in combination with another reverse transcriptase inhibitor (NRTI) usually AZT or Stavudine, and a non-nucleoside reverse transcriptase inhibitor, nevirapine or efavirenz. The four remaining patients received a combination of AZT, 3TC and lopinavir/ritonavir (LPV/r), either due to HIV-2 or HIV-1/2 dual infection, or previous exposure to single dose nevirapine. Two individuals moved onto second-line HIV-1 therapy of Tenofovir (TDF), an NRTI dually active against HBV and HIV, in combination with AZT, 3TC and LPV/r following non-suppression of HIV viraemia by first-line therapy. Patients were excluded from the study if there were any doubts as to their compliance to therapy (critical in the effectiveness of 3TC) [21]. Ethical approval was granted by the joint Gambia Government/MRC Ethics Committee. All subjects and/or legal guardians provided written, informed consent.

### Serological assays

Detection of HBsAg was performed using Determine™ HBsAg immunochromatographic test (Abbott Laboratories, USA). Samples were also tested for HBeAg and anti-HBe using an ELISA assay (DiaSorin, Salugia, Italy).

### CD4 T-cell count and HIV-1/HIV-2 viral load determination

Absolute CD4 T cell counts as well as CD4 lymphocyte proportions were determined by flow cytometry (Becton-Dickinson, Belgium) and plasma HIV-1 and HIV-2 viral load measurements made using an in-house quantitative PCR (qPCR) methodology as described previously [24].

### Biochemical assays

Liver function tests were performed using Vitros DT60 II Chemistry analyzer (Ortho-Clinical Diagnostics, Bucks, UK) according to manufacturer's instructions.

### Determination of HBV viral loads by real-time qPCR

Measurement of HBV viral load was performed using an in-house SYBR-Green method according to a previously described protocol [25], with primers HBV Taq 1 and HBV Taq 2 (Table 3). The limit of detection for the assay was 260 copies/mL and samples with viral loads higher than the top of the standard curve were diluted 1:1000 and retested.

**Table 3:** Primers designed for amplification and sequencing of HBV reverse transcriptase domain

Primer ID	Nucleotide sequences		Product size (bp)		Nucleotide positions
HBV TAQ 1	5' GTG TCT GCG GCG TTT TATCA -3'	Sense	97	DNA quantification	379-398
HBV TAQ 2	5' GAC AAA CGG GCA ACA TAC CTT-3'	Antisense			476-456
MM HBRT5	5' - ATCCTGCTGCTATGCCT - 3'	Sense	576	polymerase Amplification	410 - 426
MM HBRT6	5' - ACTTTCCAATCAATAGGCC - 3'	Antisense			986 - 968

### Amplification and sequencing of YMDD region of HBV polymerase gene

Primers for polymerase amplification were designed on the basis of conserved regions both flanking and within the reverse transcriptase region of the HBV polymerase gene, using a consensus sequence generated from reference sequences of genotypes E and A, as these are the most commonly encountered HBV genotypes in The Gambia [26,27]. The primers were synthesized by Metabion International AG (Planegg, Germany) and subsequently diluted to a concentration of 10 μM for use, according to manufacturer's instructions (Table 3).

Twenty microliters of serum was diluted 1:10 in nuclease-free water and HBV DNA extracted using the QIAamp DNA Mini Kit *(*Qiagen, Crawley, West Sussex, UK) and then re-suspended in 100 μL water. The 50-uL PCR reaction mix contained 5 μL of purified HBV DNA 25 μL HotStar Taq Master Kit (Qiagen) and 2.0 uL each of MMHBRT5 and MM HBRT6 primers. The PCR thermocycler conditions were optimised with an initial incubation at 95°C (15 mins), 40 cycles at 94°C (30 s) for denaturation, 52°C (30 s) annealing followed by extension at 72°C (1 min) which resulted in the amplification of 577-bp length of the reverse transcriptase domain covering the YMDD region. PCR products were then cleaned using the QIAquick^® ^agarose gel extraction kit (Qiagen) and sent to Macrogen, Korea for direct single-extension sequencing using the same primers (MMHBRT5 and MMHBRT6) and 3730 × l DNA analyzer (PE Applied Biosystems, Warrington, UK).

### Data management

DNA sequences were edited and assembled using SeqMan and aligned using MegAlign Lasergene software (DNAstar, Madison, WI, USA) with reference sequences of genotype E and A [accession numbers X75664 (Kou) and AM410963 respectively].

## Competing interests

The authors declare that they have no competing interests.

## Authors' contributions

Study concept and design: MM, BS, MLJ; Data analysis: IP, BS, MM. contribution to assembling of the longitudinal HIV cohort: HW, AJ, RSN, AA, SRJ, KP; responsibility for patient recruitment and treatment: TDS, KP; contributed to the drafting the manuscript: BS, MM, IP, MC, HW, TDS; and writing the paper: BS and MM. All authors read and approved the final version of the manuscript.
